# Modeling latent spatio-temporal disease incidence using penalized composite link models

**DOI:** 10.1371/journal.pone.0263711

**Published:** 2022-03-10

**Authors:** Dae-Jin Lee, María Durbán, Diego Ayma, Jan Van de Kassteele

**Affiliations:** 1 BCAM - Basque Center for Applied Mathematics, Bilbao, Bizkaia, Spain; 2 Department of Statistics, Universidad Carlos III de Madrid, Leganés, Madrid, Spain; 3 Facultad de Ciencias, Universidad Católica Norte, Antofagasta, Chile; 4 RIVM - National Institute for Public Health and the Environment, Bilthoven, Utrecht, The Netherlands; Indiana University, UNITED STATES

## Abstract

Epidemiological data are frequently recorded at coarse spatio-temporal resolutions to protect confidential information or to summarize it in a compact manner. However, the detailed patterns followed by the source data, which may be of interest to researchers and public health officials, are overlooked. We propose to use the penalized composite link model (Eilers PCH (2007)), combined with spatio-temporal P-splines methodology (Lee D.-J., Durban M (2011)) to estimate the underlying trend within data that have been aggregated not only in space, but also in time. Model estimation is carried out within a generalized linear mixed model framework, and sophisticated algorithms are used to speed up computations that otherwise would be unfeasible. The model is then used to analyze data obtained during the largest outbreak of Q-fever in the Netherlands.

## Introduction

In recent decades, the development of spatio-temporal statistical methods in Epidemiology, the cornerstone of Public Health, has been remarkable. Such development has been mainly driven by advances in geographic information systems (GISs), access to reliable health data registers, and the availability of powerful software capabilities to process and analyze large amounts of data. The methodological contributions to the analysis of spatio-temporal health data come from several interdisciplinary researchers, whose backgrounds are mostly related to Statistics, Geography, Environmental Sciences, and Epidemiology.

Within the diversity of of epidemiological research, disease mapping has attracted much interest in Public Health as it helps to visualize disease incidence of mortality risk patterns in a specific area. To this end, appropriate statistical methods have been applied to health data, which are usually recorded per spatial units, to provide smoothed disease incidences per unit. Smoothing is performed to obtain more stable and less noisy estimates of the incidence rates associated with each unit [1], which helps to determine meaningful spatial patterns. By adding the temporal dimension to this context, it is possible to examine the evolution of disease incidence in each unit, during a certain period of time (generally divided in years), but it implies a challenge for smoothing data, in terms of computational time and storage, even more if disaggregation is also needed in time. Several techniques have been proposed for the spatio-temporal smoothing of health data; most of which are developed under an empirical Bayes approach, where B-splines are used [2, 3] or a hierarchical Bayesian framework where conditional autoregressive (CAR) structures are included [4–6]. Regarding this last approach, methods that use integrated nested Laplace approximations (INLA, [7]) have recently been proposed (see [8–10]; among others).

All the works mentioned above provide smoothed estimates that change over time within a unit and across units within time interval, although each estimated value is assigned to the whole unit. Furthermore, most of them can be extended to include covariates or explanatory variables, which must have the same spatio-temporal resolution as health data. Therefore, they restrict the incorporation of population information at fine-scale and other relevant risk factors recorded at a finer resolution (see the *modifiable areal unit problem*; [11, Ch. 29]). The challenge here is not only that the map is dynamic, but the fact that, in order to obtain a better insight of the evolution of the incidence, detailed maps are needed at a finer spatial and temporal scale than the one provided (for example, grid instead of areas and weeks instead of months) while maintaining the coherence with the aggregated observed counts. To overcome the limitations of previous works, we propose the use of the *spatio-temporal penalized* composite link model(ST-PCLM) to estimate the latent distribution of spatio-temporally grouped count data. Our proposal is based on the penalized composite link model of [12], combined with a spatio-temporal P-spline methodology [13], to obtain smoothed estimates at a finer resolution from data aggregated over space and/or time. Moreover, the approach allows to include population information at fine-scale and specific random effects or further correlation structure, if necessary. This could be in the form of unstructured variation (including random effects per spatial unit) or structured variation by assuming a known variance-covariance matrix for the spatial random effects, for example using a CAR model as in [14].

As in any latent observation problem, there are infinitely possible solutions that fit the observed data. In that sense, the model is not identifiable, and the use of some prior knowledge or constraints (based on experience or common sense) is required for the problem to be well posed. Because we want to estimate a distribution, it is reasonable to expect a relatively smooth result, so we assume here that the underlying spatio-temporal process behind aggregated data is smooth and stationary (although this last hypothesis could also be relaxed by using multidimensional adaptive smoothing as in [15]). The flexibility of the model is given by the use of B-spline bases and a discrete penalty on the regression coefficients, following the P-spline methodology [16]. Smoothness is controlled by three smoothing parameters (two for the spatial dimension and one for the temporal dimension) that have to be estimated along with the overall trend at the fine scale. To perform the spatio-temporal disaggregation, the ST-PCLM uses a composition matrix that links both coarse and fine resolutions, and which is expressed as a Kronecker product of two marginal composition matrices (one acting at a spatial level and another at temporal level).

We can find several techniques that allow the spatial disaggregation of health data. For example, the use of an interpolation process from empirical Bayes estimates [17], (generalized) Poisson kriging methods [18], log-Gaussian Cox processes [19], and spatial composite link mixed models [20] have been suggested to produce a continuous smooth surface (across the study area) from regional health data. However, as far as we know, there is no appropriate model to address the problem of disaggregation of health data in both space and time (although there are some works about spatio-temporal disaggregation in a Gaussian context; see, for example, [21–25]). A common among social scientist is geographical microsimulation (see for example [26]). Geographical microsimulation models simulate populations in given geographical areas, with population characteristics as close as possible to their real counterparts. But a drawback may be that a representative sample of individuals at the fine-scale data required by those methods is not always available. The methodology presented here allows to create detailed dynamic maps for disease incidence data. Publicly available maps in aggregated form over space and time are the only information required, as well as geographical locations and time points where a finer resolution prediction is required.

## Materials and methods

### Q fever data

Q fever is a widespread zoonotic disease caused by the bacterium *Coxiella burnetii*. C. burnetii transmission to humans is mainly associated with ruminants such as cattle, sheep, and goats. During parturition or abortion of infected animals, high numbers of *C. burnetii* are shed within the amniotic fluids and the placenta. These organisms end up in the environment, where they may survive for long periods due to their resistance to heat, dryness, and many common disinfectants. Humans are often highly susceptible to the disease, and very few organisms may be necessary to cause infection. More information about this infectious disease is provided in [27].

The Southern Netherlands faced large outbreaks of human Q fever from 2007 to 2010 [28]. In this country, the local municipal health services (MHSs) are responsible for registering all confirmed diagnoses of acute Q fever. The information collected is then entered into the electronic national infectious diseases surveillance database. Due to confidentiality, these data are not publicly available and, in some instances, maybe provided in an aggregated form. In this case, the data were made available monthly at the municipality level.

**Fig 1:**
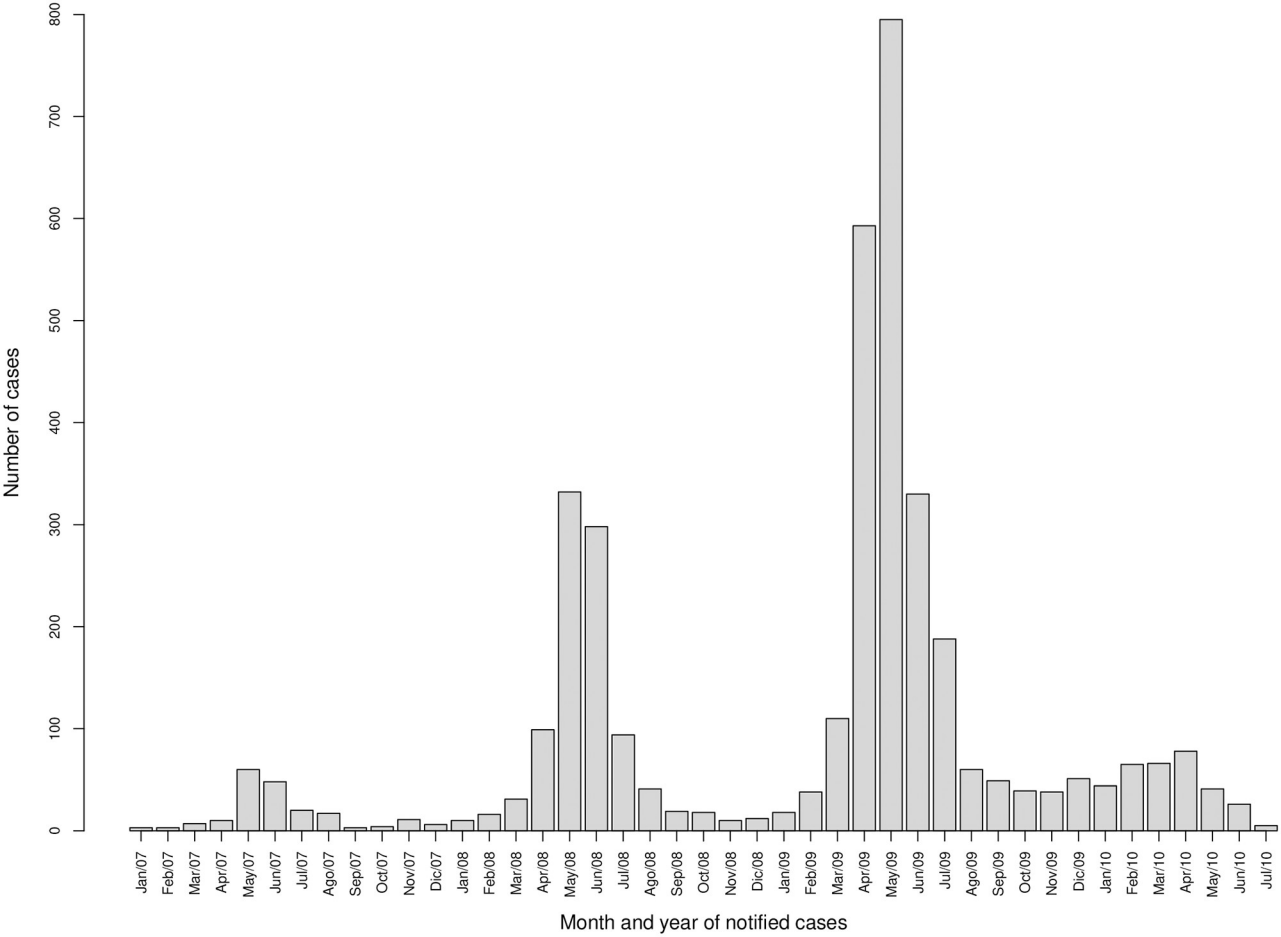
Human Q fever cases in the Netherlands grouped per months, from January 2007 to July 2010.

Fig 1 shows the temporal distribution of Q fever cases (in months) from January 2007 to July 2010. A total of 3806 acute Q fever cases were registered in this period: 192 in 2007, 980 in 2008, 2309 in 2009, and 325 in 2010. The epidemic peaks of each year were observed every spring, specifically during May. This coincides with the birth period of small ruminants (sheep and goats), a fact that was pointed out in several studies about those exceptionally large Q fever outbreaks in the Netherlands (see, for example, [28, 29]). Since the largest outbreak was observed during 2009, we have studied the distribution of Q fever incidence in that year.

**Fig 2:**
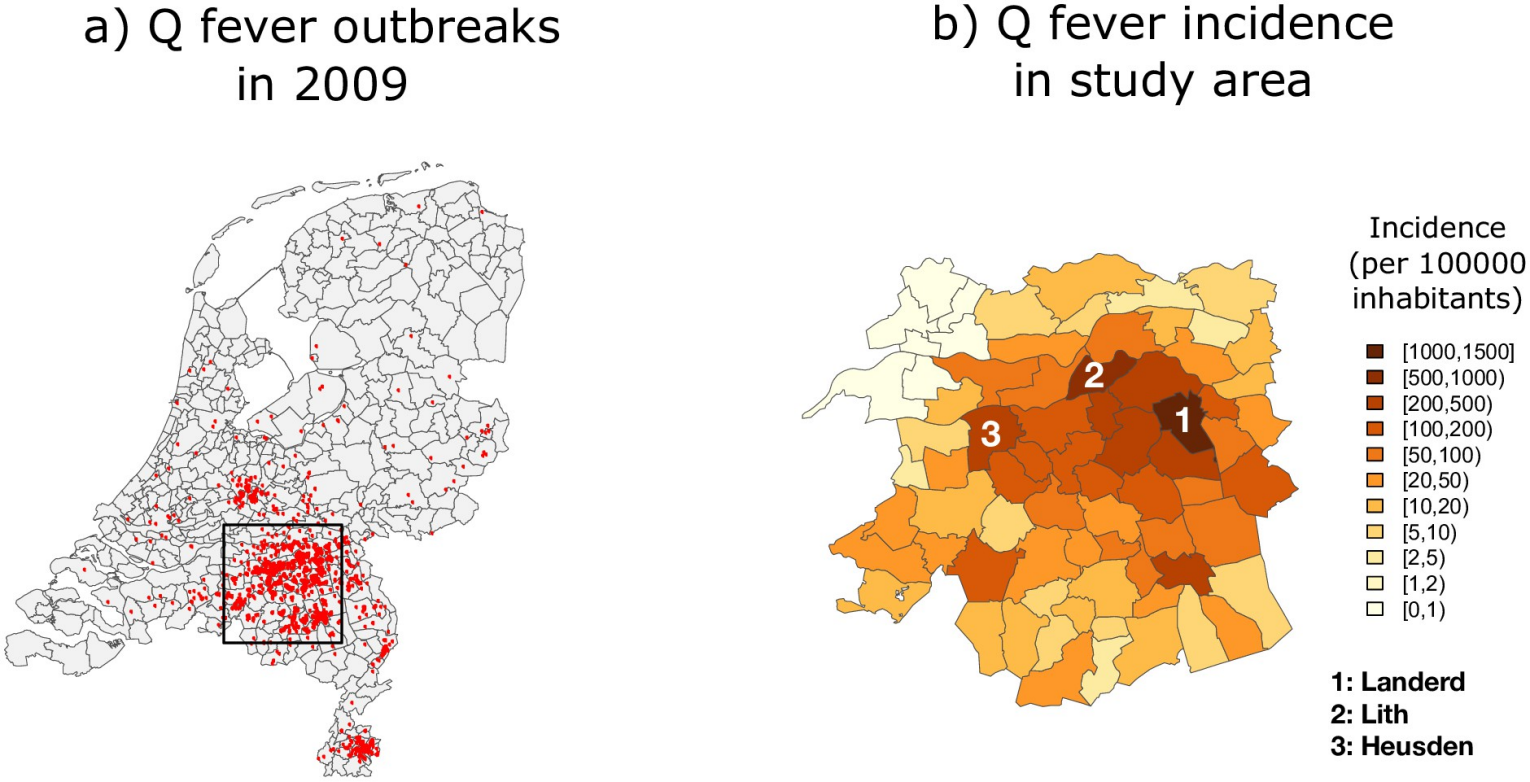
Map of human Q fever cases in the Netherlands, 2009. Left: red points indicate the residential addresses of human cases (2309 in total). Right: study area in the south of the Netherlands showing (raw) incidence (per 100000 inhabitants) of Q fever by municipality in 2009. Source: geo-referenced data are obtained from https://www.cbs.nl/en-gb/onze-diensten/open-data/statline-as-open-data/cartography. a) Q fever outbreaks in 2009. b) Q fever incidence in study area.

Fig 2a shows postal code areas affected by human Q fever (red points) in 2009. However, the number of cases was only publicly available at the municipality level. Since dairy goat and sheep farms are not evenly distributed across the Netherlands, Q-fever infection did not occur in all areas of the country. In this study we have focused on a 60 × 60km area in the south of the Netherlands (see black square in Fig 2a) with a total of 72 municipalities. The total number of Q fever cases reported in these municipalities was 1798. Taking into account the number of inhabitants in each municipality, we can calculate Q fever incidence (per 100000 inhabitants). Fig 2b shows the spatial distribution of the resulting Q fever incidence (aggregated over months in 2009), with higher incidence values observed around the municipalities of Landerd (1439.676), Lith (562.546), and Heusden (295.006). Both Figs 1 and 2 show a smooth distribution of counts in space and time which makes our initial assumption of a smooth latent distribution plausible.

### The spatio-temporal penalized composite link model

The Spatio-Temporal Penalized Composite Link Model (ST-PCLM) is the name of the model we propose for the spatio-temporal disaggregation of epidemiological data. ST-PCLM combines the penalized composite link model (PCLM) of [12] and spatio-temporal smoothing with P-splines [13]. The PCLM generalizes the model proposed by [30] and allows the estimation of a smooth trend, from grouped data, at a finer resolution. The idea behind it is as follows:

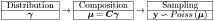


We observe data **y** from a Poisson distribution with mean ***μ***, then we assume that ***μ*** is a composition of the ungrouped distribution ***γ***. The approach assumes that the underlying (ungrouped) distribution is smooth, but otherwise let the data determine their shape.

As PCLM applies to the unidimentional scenario, we develop it in the spatio-temporal context. Then, we have included the ideas presented in [13] to reformulate the new spatio-temporal PCLM under a generalized linear mixed model (GLMM) framework, i.e., ST-PCLM. Hereafter, we will show the development of the ST-PCLM and a computationally efficient way to estimate the ST-PCLM parameters.

#### The model

Let *y*_*it*_, *i* = 1, …, *n*, *t* = 1, …, *T*_a_, denote count data recorded over *n* non-overlapping spatial units *v*_*i*_, which constitute the area of interest, at *T*_a_ time periods. Suppose that we want to estimate the latent distribution of the vector of counts at a spatio-temporal support that is a refinement of the original one. The fine support is determined by three coordinates: ***x***_1_ = (*x*_11_, …, *x*_1*m*_)′ and ***x***_2_ = (*x*_21_, …, *x*_2*m*_)′, with *m* > *n*, which represent the longitude and latitude coordinates of the spatial refinement, respectively; and $x_{3}={\left(x_{31},...,x_{3T_{\text{f}}}\right)}^{\prime }$, with *T*_f_ > *T*_a_, which represents the coordinates of the temporal refinement. We assume that the spatial refinement remains fixed at each instant of time in ***x***_3_, but the method can easily be extended to relax this assumption. Assuming that ***y*** is Poisson distributed with mean vector ***μ***, the ST-PCLM is given by:

$$\begin{array}{c} \mu =C_{\text{st}}\gamma_{\text{f}}=C_{\text{st}}\text{exp}\left\{f_{\text{st}}\left(x_{1},x_{2},x_{3}\right)\right\}\text{,} \end{array}$$ (1)

where ***γ***_f_ denotes the fine-scale latent mean, **C**_st_ is the composition matrix that describes how these latent means are combined to yield ***μ***, and *f*_st_(***x***_1_, ***x***_2_, ***x***_3_) represents the fine-scale spatio-temporal trend. Several approaches can be used to model that function, e.g.: Gaussian random fields, spatio-temporal kriging or penalized splines, among others. We propose the last option since smoothness might be a reasonable property to ask for: if we are estimating an unknown distribution, it seems natural to assume smoothness since we do not know much about the underlying process (the use of the other techniques mentioned is out of the scope of this paper and will be subject of further work). We assume that the non-separable function *f*_st_(***x***_1_, ***x***_2_, ***x***_3_) may be represented by the product of simpler functions $f_{x_{1}}\left(x_{1}\right)$, $f_{x_{2}}\left(x_{2}\right)$, and $f_{x_{3}}\left(x_{3}\right)$. Therefore, the basis representation of the function would be given by:

$$\begin{array}{c} f_{\text{st}}\left(x_{1},x_{2},x_{3}\right)=B_{\text{st}}\theta =\left\{B_{3}⊗(B_{2}□B_{1})\right\}\theta \text{,} \end{array}$$ (2)

where **B**_1_ = **B**(***x***_1_), **B**_2_ = **B**(***x***_2_), and **B**_3_ = **B**(***x***_3_) are univariate B-spline bases [16] of dimensions *m* × *c*_1_, *m* × *c*_2_ and *T*_f_ × *c*_3_ respectively. The matrix operators ⊗ and □ on the right-hand side of Eq (2) represent Kronecker and Box products (row tensor products), respectively [31] (if the spatial refinement changes over time, Box product would be used instead of Kronecker product). To achieve smoothness, we use an anisotropic penalty (based on second order differences constructed separately for each independent variable) over the vector of regression coefficients ***θ***. The form of this matrix is as follows:

$$\begin{array}{c} P_{\text{st}}=\lambda_{1}I_{c_{3}}⊗I_{c_{2}}⊗P_{1}+\lambda_{2}I_{c_{3}}⊗P_{2}⊗I_{c_{1}}+\lambda_{3}P_{3}⊗I_{c_{2}}⊗I_{c_{1}}\text{,} \end{array}$$ (3)

where $I_{c_{d}}$ denotes an identity matrix of dimension *c*_*d*_ × *c*_*d*_, λ_*d*_ is the smoothing parameter that controls the amount of smoothing along the coordinate ***x***_*d*_, and $P_{d}=D_{d}^{\prime }D_{d}$ is the marginal penalty matrix based on **D**_*d*_, which computes *q*_*d*_-th differences, i.e., $\Delta^{q_{d}}\theta =D_{d}\theta$, for *d* = 1, 2, 3. As we mentioned earlier, the matrix in Eq (3) has an anisotropic property in the sense that allows a different amount of smoothing for each coordinate.

Since we are assuming that the spatial refinement is fixed over the temporal refinement, the composition matrix **C**_st_ in model (1) is obtained as:

$$\begin{array}{c} C_{\text{st}}=C_{\text{t}}⊗C_{\text{s}}\text{,} \end{array}$$ (4)

where **C**_s_ and **C**_t_ are the spatial and temporal composition matrices of dimensions *n* × *m* and *T*_a_ × *T*_f_, respectively. The structure of these matrices depends on the type and level of aggregation. For example, if we want to estimate the latent distribution at a fine spatial grid (over a study area), the entries of the spatial composition matrix will be:

$$\begin{array}{c} {\left[C_{\text{s}}\right]}_{ij}=\{\begin{array}{cc} 1 & \text{if}\;\left(x_{1j},x_{2j}\right)\;\text{belongs}\;\text{to}\;\text{unit}\;v_{i}\\ 0 & \text{otherwise} \end{array} \end{array}$$ (5)

where (*x*_1*j*_, *x*_2*j*_) are the cell centroid coordinates of the fine grid, for *i* = 1, …, *n* and *j* = 1, …, *m*. Another option to construct **C**_s_ is to consider its entries as the area proportions that each grid cell shares with a specific unit. The temporal composition matrix **C**_t_ is used to disaggregate coarse time intervals into detailed time periods (for example, from years or trimesters to months, weeks, or days). In our application, we show the structure that **C**_t_ will have, specifically for our purposes. Note that if **C**_s_ = **I**_*n*_, $C_{\text{t}}=I_{T_{\text{a}}}$, and the unit centroids are used as spatial coordinates, the presented methodology is reduced to the Poisson version of the proposal given by [13] for smoothing of spatio-temporal count data.

When spatial data are recorded over a coarse rectangular grid, an appropriate definition for **B**_st_ in Eq (2) is **B**_st_ = **B**_3_ ⊗ (**B**_2_ ⊗ **B**_1_), where the spatial refinement correspond to the cell centroid coordinates of a fine grid. The penalty matrix **P**_st_ in Eq (3) is still valid in this context, because its definition is independent of data structure. Moreover, the spatial composition matrix would be given by **C**_s_ = **C**_2_ ⊗ **C**_1_, where each **C**_*d*_ is constructed according to the disaggregation of the coarse grid cells into small ones, for *d* = 1, 2. Although the Kronecker structure in (4) is a computational advantage, our model can cope with more complex situations where the spatial disaggregation changes from one time point to another (for example, census tracks changes along the years) by simply changing the structure of **C**_st_.

#### Mixed model representation

Now, we show how to incorporate fine-scale population information into the model (1). To do so, we must reformulate the model as a Generalized Linear Mixed Model (GLMM) by following the proposal of [13] as it is briefly described below.

In the P-splines literature, [13] show a nice representation of the spatio-temporal trend as a mixed model. Following their approach, the term *f*_st_(***x***_1_, ***x***_2_, ***x***_3_) in model (2) can be rewritten as *f*_st_(***x***_1_, ***x***_2_, ***x***_3_) = **B**_st_ ***θ*** = ***x*** **β** + **Z** ***α***. Thus, we have:

$$\begin{array}{c} \mu =C_{\text{st}}\gamma_{\text{f}}=C_{\text{st}}\left\{\text{exp}\left(X\beta +Z\alpha +\text{log}\left(e_{\text{f}}\right)\right)\right\}\text{,}\;\text{with}\;\alpha ∼N(0,G)\text{,} \end{array}$$ (6)

where **X** and **Z** are fixed and random effects matrices, and ***β*** and ***α*** are their associated coefficients, respectively. Random effects have covariance matrix **G** that depends on the smoothing parameters. The model includes an offset term log(***e***_f_) that allows the analysis of mortality or incidence rates instead of counts. The vector ***e***_f_ could be, for example, fine-scale population information or expected number of deaths. If we only have the offset term at the coarse scale, i.e., $\text{log}\left(e\right)={\left(\text{log}\left(e_{11}\right),...,\text{log}\left(e_{n1}\right),...,\text{log}\left(e_{1T_{1}}\right),....,\text{log}\left(e_{nT_{\text{a}}}\right)\right)}^{\prime }$, a naive approach to estimate ***e***_f_ assumes that the elements of ***e*** are evenly distributed throughout the fine resolution. Therefore, we can compute these naive estimates as ${\hat{e}}_{\text{naive}}=C_{\text{st}}^{-}e$, where $C_{\text{st}}^{-}$ denotes the Moore-Penrose inverse of **C**_st_.

The construction of the matrices **X**, **Z**, and **G** in model (6) are obtained by using the singular value decomposition (SVD) of each discrete penalty matrix **P**_*d*_ in Eq (3), for *d* = 1, 2, 3. The mixed model matrices are given by:

$$\begin{array}{c} \begin{array}{cc} X & =X_{3}⊗(X_{2}□X_{1})\text{,}\\ Z & =[Z_{3}⊗(X_{2}□X_{1}):X_{3}⊗(Z_{2}□X_{1}):X_{3}⊗(X_{2}□Z_{1}):\\ & Z_{3}⊗(Z_{2}□X_{1}):Z_{3}⊗(X_{2}□Z_{1}):X_{3}⊗(Z_{2}□Z_{1}):Z_{3}⊗(Z_{2}□Z_{1})]\text{,} \end{array} \end{array}$$ (7)

where **X**_*d*_ = **B**_*d*_ **U**_*dn*_ and **Z**_*d*_ = **B**_*d*_ **U**_*ds*_ are constructed from the matrices of singular vectors corresponding to null and non-null singular values of the SVD of **P**_*d*_, **U**_*dn*_ and **U**_*ds*_, respectively, for *d* = 1, 2, 3. Denoting ${\tilde{\Sigma }}_{d}$ as the diagonal matrix with the non-null singular values of the SVD of **P**_*d*_ in the main diagonal, the inverse of the covariance matrix **G** becomes the block-diagonal matrix:

$$\begin{array}{cc} G^{-1}=\text{blockdiag} & (\lambda_{3}F_{3u},\lambda_{2}F_{2u},\lambda_{1}F_{1u},\\ & \lambda_{2}F_{22}+\lambda_{3}F_{32},\lambda_{1}F_{12}+\lambda_{3}F_{31},\lambda_{1}F_{11}+\lambda_{2}F_{21},\\ & \lambda_{1}F_{1t}+\lambda_{2}F_{2t}+\lambda_{3}F_{3t})\text{,} \end{array}$$ (8)

where:

$$\begin{array}{ccc} F_{1u}=I_{q_{3}}⊗I_{q_{2}}⊗{\tilde{\Sigma }}_{1}, & F_{2u}=I_{q_{3}}⊗{\tilde{\Sigma }}_{2}⊗I_{q_{1}}, & F_{3u}={\tilde{\Sigma }}_{3}⊗I_{q_{2}}⊗I_{q_{1}},\\ F_{11}=I_{q_{3}}⊗I_{c_{2}-q_{2}}⊗{\tilde{\Sigma }}_{1}, & F_{12}=I_{c_{3}-q_{3}}⊗I_{q_{2}}⊗{\tilde{\Sigma }}_{1}, & F_{21}=I_{q_{3}}⊗{\tilde{\Sigma }}_{2}⊗I_{c_{1}-q_{1}},\\ F_{22}=I_{c_{3}-q_{3}}⊗{\tilde{\Sigma }}_{2}⊗I_{q_{1}}, & F_{31}={\tilde{\Sigma }}_{3}⊗I_{q_{2}}⊗I_{c_{1}-q_{1}}, & F_{32}={\tilde{\Sigma }}_{3}⊗I_{c_{2}-q_{2}}⊗I_{q_{1}},\\ F_{1t}=I_{c_{3}-q_{3}}⊗I_{c_{2}-q_{2}}⊗{\tilde{\Sigma }}_{1}, & F_{2t}=I_{c_{3}-q_{3}}⊗{\tilde{\Sigma }}_{2}⊗I_{c_{1}-q_{1}}, & F_{3t}={\tilde{\Sigma }}_{3}⊗I_{c_{2}-q_{2}}⊗I_{c_{1}-q_{1}}. \end{array}$$

If data are spatially recorded over a rectangular coarse grid, the corresponding mixed model matrices are obtained as in Eq (7), but replacing the Box products □ by Kronecker products ⊗. The formulation of **G**^−1^ remains the same.

#### Parameter estimation

Once the ST-PCLM defined in (6) is in the GLMM framework, it is possible to estimate its parameters. This estimation procedure was presented by [20] in a spatial disaggregation context, and it involves two interrelated stages: (a) estimation of fixed coefficients and random effects (***β*** and ***α***); and (b) estimation of smoothing parameters (λ_1_, λ_2_, and λ_3_). The penalized quasi-likelihood (PQL) methods of [32] are used for stage (a), and the restricted (or residual) maximum likelihood (REML, [32, 33]) is used for stage (b) as a numerical optimization criterion for smoothing parameter selection. Technical details are provided in [20] and, thus, we only describe here the necessary results.

For given values of λ_1_, λ_2_, and λ_3_, the estimation of the fixed and random effects coefficients of the model (6) are obtained by maximizing the following approximate penalized log-likelihood:

$$\begin{array}{c} y^{\prime }\text{log}\left(\mu \right)-1^{\prime }\mu -\frac{1}{2}\alpha^{\prime }G^{-1}\alpha \text{,} \end{array}$$ (9)

where **1** denotes a vector of ones of length *n* ⋅ *T*_a_ and ***μ*** = **C**_st_ ***γ***_f_, with ***γ***_f_ = ***e***_f_ * exp(**X** ***β*** + **Z** ***α***). Differentiation of Eq (9) with respect to ***β*** and ***α*** leads to the score equations:

$$\begin{array}{c} \begin{array}{cc} {\overset{˘}{X}}^{\prime }(y-\mu ) & =0\text{,}\\ {\overset{˘}{Z}}^{\prime }(y-\mu ) & =G^{-1}\alpha \text{,} \end{array} \end{array}$$ (10)

where $\overset{˘}{X}=W^{-1}C_{\text{st}}\Gamma X$ and $\overset{˘}{Z}=W^{-1}C_{\text{st}}\Gamma Z$ are called ‘working’ mixed model matrices, since **W** = diag(***μ***) and **Γ** = diag(***γ***_f_) change during the estimation procedure. Defining the working vector as:

$$\begin{array}{c} z=\overset{˘}{X}\beta +\overset{˘}{Z}\alpha +W^{-1}(y-\mu )\text{,} \end{array}$$

the solution of the score equations in (10) via Fisher scoring algorithm is expressed as the iterative solution of the system:

$$\begin{array}{c} {}[\begin{array}{cc} {\overset{˘}{X}}^{\prime }W\overset{˘}{X} & {\overset{˘}{X}}^{\prime }W\overset{˘}{Z}G\\ {\overset{˘}{Z}}^{\prime }W\overset{˘}{X} & I+{\overset{˘}{Z}}^{\prime }W\overset{˘}{Z}G \end{array}][\begin{array}{c} \beta \\ b \end{array}]=[\begin{array}{c} {\overset{˘}{X}}^{\prime }Wz\\ {\overset{˘}{Z}}^{\prime }Wz \end{array}]\text{,} \end{array}$$ (11)

where ***b*** = **G**^−1^ ***α***. This yields to a modified version of the standard mixed model estimators:

$$\begin{array}{c} \hat{\beta }={\left({\overset{˘}{X}}^{\prime }V^{-1}\overset{˘}{X}\right)}^{-1}{\overset{˘}{X}}^{\prime }V^{-1}z\text{,} \end{array}$$ (12)

$$\begin{array}{cc} \hat{\alpha } & =G{\overset{˘}{Z}}^{\prime }V^{-1}\left(z-\overset{˘}{X}\hat{\beta }\right)\\ & =G{\overset{˘}{Z}}^{\prime }Nz\text{,} \end{array}$$ (13)

where:

$$\begin{array}{c} V=W^{-1}+\overset{˘}{Z}G{\overset{˘}{Z}}^{\prime }\text{,} \end{array}$$

$$\begin{array}{c} N=V^{-1}-V^{-1}\overset{˘}{X}{\left({\overset{˘}{X}}^{\prime }V^{-1}\overset{˘}{X}\right)}^{-1}{\overset{˘}{X}}^{\prime }V^{-1}. \end{array}$$ (15)

Conditioning on the estimates (12) and (13), the smoothing parameters λ_1_, λ_2_, and λ_3_ can be estimated by maximizing the approximate REML (see [32, Eq. 13]):

$$\begin{array}{cc} l^{*}\left(V\right) & =\frac{1}{2}\text{log}|V|-\frac{1}{2}\text{log}\left|{\overset{˘}{X}}^{\prime }V^{-1}\overset{˘}{X}\right|-\frac{1}{2}{\left(z-\overset{˘}{X}\hat{\beta }\right)}^{\prime }V^{-1}\left(z-\overset{˘}{X}\hat{\beta }\right)\\ & =\frac{1}{2}\text{log}|V|-\frac{1}{2}\text{log}\left|{\overset{˘}{X}}^{\prime }V^{-1}\overset{˘}{X}\right|-\frac{1}{2}z^{\prime }\left(V^{-1}-V^{-1}\overset{˘}{X}{\left({\overset{˘}{X}}^{\prime }V^{-1}\overset{˘}{X}\right)}^{-1}{\overset{˘}{X}}^{\prime }V^{-1}\right)z\text{,} \end{array}$$ (16)

where λ_1_, λ_2_, and λ_3_ are involved in **V** through **G**. Therefore, optimal estimates for the ST-PCLM parameters are obtained by iteration between Eqs (12), (13) and (16), until convergence.

Once the ST-PCLM parameter estimates at convergence are obtained, we can derive standard errors for $\hat{\eta }=X\hat{\beta }+Z\hat{\alpha }$ by using the Bayesian approximation of the variance-covariance matrix for ${\left(\hat{\beta },\hat{\alpha }\right)}^{\prime }$ (see [34] for further details). Thus, the approximate standard errors for $\hat{\eta }$ are obtained by taking the square root of $V\text{ar}(\hat{\eta })$, which is obtained as:

$$\begin{array}{c} V\text{ar}\left(\hat{\eta }\right)=\text{diag}(\left[X:Z\right]{\left[\begin{array}{cc} {\overset{˘}{X}}^{\prime }W\overset{˘}{X} & {\overset{˘}{X}}^{\prime }W\overset{˘}{Z}\\ {\overset{˘}{Z}}^{\prime }W\overset{˘}{X} & {\overset{˘}{Z}}^{\prime }W\overset{˘}{Z}+G^{-1} \end{array}\right]}^{-1}{\left[X:Z\right]}^{\prime })\text{.} \end{array}$$

We should note that the estimation procedure described above can be computationally inefficient if we are working with large datasets and the goal is to obtain estimates at a very fine scale. Furthermore, the direct creation of the matrices **C**_sf_, **X**, and **Z** in model (7) can easily lead to storage problems. In the next Sections, we will provide solutions for the ST-PCLM estimation in terms of storage and efficiency (these solutions were not available in [20]).

#### Fast algorithm for spatio-temporal penalized composite link models

In order to efficiently compute estimates of the smoothing parameters, we have adapted the SAP (Separation of Anisotropic Penalties) algorithm of [35] to the ST-PCLM context. To avoid possible storage problems, we have used the so-called GLAM (*Generalized Linear Array Methods*, [31, 36]) in the ST-PCLM setting. These methods also provide an efficient way to compute the matrix of cross-products required for the SAP algorithm, easing the computation time of model estimation.

Under the ST-PCLM approach and conditioning on the estimates given in Eqs (12) and (13), we can estimate λ_1_, λ_2_, and λ_3_ by numerical maximization of the approximate REML in Eq (16). The usual algorithms used to approximate the solutions, such as the one proposed by [37] (extended to the generalized case by [38]), can only deal with situations in which the variance-covariance matrix is linear on the variance parameters. In the case of spatio-temporal models with anisotropic penalties, regression parameters are affected by more than one smoothing parameter, and so standard approaches can’t be used, since the corresponding variance-covariance matrix does not have the required form. [35] proposed the SAP algorithm which provides a numerical solution to REML estimates, but it is able to deal with models that have a precision matrix for the random effect vector that is linear in the inverse of the variance parameters. These precision matrices are common when penalized smooth models with anisotropic penalties are reformulated as (generalized) linear mixed models, where the smoothing parameters are seen as ratios of variance components, i.e., $\lambda_{d}=\frac{\phi }{\tau_{d}^{2}}$, for *d* = 1, 2, 3. Since we are working under a Poisson framework, the dispersion parameter, *ϕ*, is equal to 1. Thus, the problem is reduced to obtain estimates for the variance components $\tau_{1}^{2}$, $\tau_{2}^{2}$, and $\tau_{3}^{2}$. The reformulation of the SAP algorithm for the ST-PCLM is described below.

Following [35] we can derive closed-form expressions, from the approximate REML, for the variance components $\tau_{d}^{2}$, for *d* = 1, 2, 3. These estimates are given by:

$$\begin{array}{c} {\hat{\tau }}_{d}^{2}=\frac{{\hat{\alpha }}^{\prime }\Lambda_{d}\hat{\alpha }}{{\text{ed}}_{d}}\text{,} \end{array}$$ (17)

where:

$$\begin{array}{c} {\text{ed}}_{d}=\text{trace}({\overset{˘}{Z}}^{\prime }N\overset{˘}{Z}G\frac{\Lambda_{d}}{\tau_{d}^{2}}G)\text{,} \end{array}$$ (18)

with **N** defined in Eq (15), and

$$\begin{array}{c} \Lambda_{1}=\text{blockdiag}(0_{q_{1}q_{2}\left(c_{3}-q_{3}\right)},0_{q_{1}q_{3}\left(c_{2}-q_{2}\right)},F_{1u},0_{q_{1}\left(c_{2}-q_{2}\right)\left(c_{3}-q_{3}\right)},F_{12},F_{11},F_{1t})\text{,}\\ \Lambda_{2}=\text{blockdiag}(0_{q_{1}q_{2}\left(c_{3}-q_{3}\right)},F_{2u},0_{q_{2}q_{3}\left(c_{1}-q_{1}\right)},F_{22},0_{q_{2}\left(c_{1}-q_{1}\right)\left(c_{3}-q_{3}\right)},F_{21},F_{2t})\text{,}\\ \Lambda_{3}=\text{blockdiag}(F_{3u},0_{q_{1}q_{3}\left(c_{2}-q_{2}\right)},0_{q_{2}q_{3}\left(c_{1}-q_{1}\right)},F_{32},F_{31},0_{q_{3}\left(c_{1}-q_{1}\right)\left(c_{2}-q_{2}\right)},F_{3t})\text{.} \end{array}$$

The non-null submatrices of each **Λ**_*d*_ were previously defined in Section. Here the inverse of the covariance matrix **G** in model (6) is written in terms of $\tau_{d}^{2}$’s and can be decomposed as $G^{-1}=\frac{1}{\tau_{1}^{2}}\Lambda_{1}+\frac{1}{\tau_{2}^{2}}\Lambda_{2}+\frac{1}{\tau_{3}^{2}}\Lambda_{3}$, where the capital lambdas are defined above. The SAP algorithm for the ST-PCLM parameter estimation is given in Algorithm 1, which is an adaptation of the algorithm provided in [35, p. 945].

**Algorithm 1** SAP algorithm for the ST-PCLM parameters estimation

**Require**: Convergence tolerances *ν*_1_ and *ν*_2_ (e.g., 1 × 10^−6^) and maximum number of iterations maxit_1_ and maxit_2_ (e.g., 100).

1: Set initial values for the mixed model coefficients ***β*** and ***α***, and the variance components $\tau_{1}^{2}$, $\tau_{2}^{2}$, and $\tau_{3}^{2}$ (for example, ${\hat{\beta }}^{\left(0\right)}=0$ with length *q*_1_ *q*_2_ *q*_3_, ${\hat{\alpha }}^{\left(0\right)}=0$ with length (*c*_1_ *c*_2_ *c*_3_−*q*_1_ *q*_2_ *q*_3_), and ${\hat{\tau }}_{1}^{2(0)}={\hat{\tau }}_{2}^{2(0)}={\hat{\tau }}_{3}^{2(0)}=1$). Set *k* = 0.

2: **for** 1 **to** maxit_1_ **do**

3:  Given the current estimates for the mixed model coefficients, construct the matrix of weights **W** and the working vector **z** as follows:

$$\begin{array}{cc} \Gamma & =\text{diag}\left({\hat{\gamma }}_{\text{f}}^{\left(k\right)}\right)\text{,}\;\text{with}\;{\hat{\gamma }}_{\text{f}}^{\left(k\right)}=\text{exp}\left(X{\hat{\beta }}^{\left(k\right)}+Z{\hat{\alpha }}^{\left(k\right)}+\text{log}\left(e_{\text{f}}\right)\right)\text{;}\\ W & =\text{diag}\left({\hat{\mu }}^{\left(k\right)}\right)\text{,}\;\text{with}\;{\hat{\mu }}^{\left(k\right)}=C_{\text{st}}{\hat{\gamma }}_{\text{f}}^{\left(k\right)}\text{;}\\ z & =\overset{˘}{X}{\hat{\beta }}^{\left(k\right)}+\overset{˘}{Z}{\hat{\alpha }}^{\left(k\right)}+W^{-1}\left(y-{\hat{\mu }}^{\left(k\right)}\right)=W^{-1}C\Gamma {\hat{\eta }}^{\left(k\right)}+W^{-1}\left(y-{\hat{\mu }}^{\left(k\right)}\right)\text{,} \end{array}$$

with ${\hat{\eta }}^{\left(k\right)}=X{\hat{\beta }}^{\left(k\right)}+Z{\hat{\alpha }}^{\left(k\right)}$.

4:  **for** 1 **to** maxit_2_ **do**

5:   Given the current estimates for the variance components, obtain new estimates for ***β*** and ***α*** by solving the system in Eq (11). The resulting estimates are denoted as ${\hat{\beta }}^{\left(k+1\right)}$ and ${\hat{\alpha }}^{\left(k+1\right)}$, respectively.

6:   Obtain new estimates for the variance components using Eq (17). The resulting estimates are denoted as ${\hat{\tau }}_{d}^{2(k+1)}$, for *d* = 1, 2, 3.

7:   Compare new variance component estimates with the previous ones, using the following convergence criterion:

$$\begin{array}{c} \frac{\sum_{d=1}^{3}\left|{\hat{\tau }}_{d}^{2(k+1)}-{\hat{\tau }}_{d}^{2(k)}\right|}{3}\leq \nu_{1}\text{.} \end{array}$$

8:   If the convergence tolerance is achieved, **break**, otherwise set ${\hat{\tau }}_{d}^{2(k)}={\hat{\tau }}_{d}^{2(k+1)}$ and repeat steps 5, 6, and 7 until convergence.

9:  **end for**

10:  Compute a new estimate for the fine-scale smooth trend vector using the fixed and random effects estimates obtained in the last iteration of step 5. The resulting vector is denoted as ${\hat{\eta }}^{\left(k+1\right)}$. Compare the new estimate with the previous one, using the following convergence criterion:

$$\begin{array}{c} \frac{\|{\hat{\eta }}^{\left(k+1\right)}-{\hat{\eta }}^{\left(k\right)}{\|}^{2}}{\|{\hat{\eta }}^{\left(k+1\right)}{\|}^{2}}\leq \nu_{2}\text{.} \end{array}$$

11:  If the convergence tolerance is achieved, **break**, otherwise set *k* = *k* + 1 and repeat steps 2 to 11 until convergence.

12: **end for**

Although we have suggested setting the maximum number of iterations to 100, our experience is that the number of iterations needed to achieve convergence is much smaller. In general, we have observed that the maximum number of iterations to obtain optimal variance components is greater than the number of iterations to obtain optimal estimates for the linear predictor. In the analysis of Q-fever data, convergence was reached after 30 iterations.

We should note that we can efficiently compute the trace in Eq (18) by taking into account that **G** **Λ**_*d*_ **G** is a diagonal matrix. Thus, we only have to compute the diagonal of ${\overset{˘}{Z}}^{\prime }N\overset{˘}{Z}$ to obtain this trace. From [39, Eq. (5.3)], we have that:

$$\begin{array}{c} {\overset{˘}{Z}}^{\prime }N=\left[0_{\left(c_{1}c_{2}c_{3}-q_{1}q_{2}q_{3}\right)\times q_{1}q_{2}q_{3}}:I_{\left(c_{1}c_{2}c_{3}-q_{1}q_{2}q_{3}\right)}\right]{\left[\begin{array}{cc} {\overset{˘}{X}}^{\prime }W\overset{˘}{X} & {\overset{˘}{X}}^{\prime }W\overset{˘}{Z}G\\ {\overset{˘}{Z}}^{\prime }W\overset{˘}{X} & I+{\overset{˘}{Z}}^{\prime }W\overset{˘}{Z}G \end{array}\right]}^{-1}\left[\overset{˘}{X}:\overset{˘}{Z}\right]W\text{.} \end{array}$$

Therefore, the diagonal elements of matrix ${\overset{˘}{Z}}^{\prime }N\overset{˘}{Z}$ are obtained by the column-wise addition of:

$$\begin{array}{c} {\left(\left[0_{\left(c_{1}c_{2}c_{3}-q_{1}q_{2}q_{3}\right)\times q_{1}q_{2}q_{3}}:I_{\left(c_{1}c_{2}c_{3}-q_{1}q_{2}q_{3}\right)}\right]{\left[\begin{array}{cc} {\overset{˘}{X}}^{\prime }W\overset{˘}{X} & {\overset{˘}{X}}^{\prime }W\overset{˘}{Z}G\\ {\overset{˘}{Z}}^{\prime }W\overset{˘}{X} & I+{\overset{˘}{Z}}^{\prime }W\overset{˘}{Z}G \end{array}\right]}^{-1}\right)}^{\prime }⊙[\begin{array}{c} {\overset{˘}{X}}^{\prime }W\overset{˘}{Z}\\ {\overset{˘}{Z}}^{\prime }W\overset{˘}{Z} \end{array}]\text{.} \end{array}$$

An advantage of using the adapted SAP algorithm provided above is that we can directly compute the effective dimension (ED) of model (6). This model complexity measure is given by:

$$\begin{array}{c} \text{ED}=q_{1}q_{2}q_{3}+\sum_{d=1}^{3}{\text{ed}}_{d}\text{,} \end{array}$$ (19)

where each ed_*d*_ is computed from Eq (18). The first term on the right-hand side of Eq (19) corresponds to the dimension of the unpenalized (or fixed) part, whereas the second corresponds to the unpenalized (or random) part of the fitted model. To visualize the later statement, note that:

$$\begin{array}{c} \sum_{d=1}^{3}{\text{ed}}_{d}=\text{trace}(\sum_{d=1}^{3}{\overset{˘}{Z}}^{\prime }N\overset{˘}{Z}G\frac{\Lambda_{d}}{\tau_{d}^{2}}G)=\text{trace}({\overset{˘}{Z}}^{\prime }N\overset{˘}{Z}G)=\text{trace}(\overset{˘}{Z}G{\overset{˘}{Z}}^{\prime }N)\text{,} \end{array}$$

where $\overset{˘}{Z}G{\overset{˘}{Z}}^{\prime }N$ is the ‘hat matrix’ [40] of the unpenalized part of the fitted ST-PCLM (see Eq (13)).

Note that the matrix cross-products ${\overset{˘}{X}}^{\prime }W\overset{˘}{X}$, ${\overset{˘}{X}}^{\prime }W\overset{˘}{Z}$, and ${\overset{˘}{Z}}^{\prime }W\overset{˘}{Z}$ (and its transpose) are involved in the computation of the variance component estimates. They also appear in the estimation of fixed and random effects coefficients, which are obtained by solving the system in Eq (11). These and other required matrix cross-products can be efficiently computed by adapting the so-called GLAM methods to the ST-PCLM setting, which we will show in the next Section.

#### GLAM methods for spatio-temporal penalized composite link models

When we deal with estimating latent trends in multiple dimensions, we are susceptible to problems with storage and computational burden. In the case of data arranged in multidimensional grids, these problems can be overcome using the GLAM methods developed in [31, 36]. These methods are designed to avoid direct computation of matrix cross-products where Kronecker operations are involved, by using sequences of nested matrix operations. In this Section, we show the use of these methods in the ST-PCLM context.

First consider the matrix-by-vector products **X** ***β***, **Z** ***α***, and **C**_st_ ***γ***_f_ that appear in line 3 of Algorithm 1. These expressions can be computed as:

$$\begin{array}{cc} X\beta & \equiv \rho \{X_{3},\rho \left(R_{1},\tilde{Q}\right)\}\text{,}\\ Z\alpha & \equiv [\rho \left\{Z_{3},\rho \left(R_{1},{\tilde{A}}_{1}\right)\right\}:\rho \left\{X_{3},\rho \left(R_{2},{\tilde{A}}_{2}\right)\right\}:\rho \left\{X_{3},\rho \left(R_{3},{\tilde{A}}_{3}\right)\right\}:\rho \left\{Z_{3},\rho \left(R_{2},{\tilde{A}}_{4}\right)\right\}:\\ & \;\rho \left\{Z_{3},\rho \left(R_{3},{\tilde{A}}_{5}\right)\right\}:\rho \left\{X_{3},\rho \left(R_{4},{\tilde{A}}_{6}\right)\right\}:\rho \left\{Z_{3},\rho \left(R_{4},{\tilde{A}}_{7}\right)\right\}]\text{,}\\ C_{\text{st}}\gamma_{\text{f}} & \equiv \rho \{C_{\text{t}},\rho \left(C_{\text{s}},\tilde{\Gamma }\right)\}\text{,} \end{array}$$

with $R_{1}=G\left(X_{2},X_{1}\right)$, $R_{2}=G\left(Z_{2},X_{1}\right)$, $R_{3}=G\left(X_{2},Z_{1}\right)$, $R_{4}=G\left(Z_{2},Z_{1}\right)$, where *ρ* and G denote the rotated H- transform and the row-tensor product, respectively, defined in the S1 File. The symbol ≡ means that both sides have the same elements but in a different order. The matrices $\tilde{Q}$, $\tilde{\Gamma }$, and ${\tilde{A}}_{k}$, for *k* = 1, …, 7, are arrangements of the vectors ***β***, ***γ***, and ***α***_*k*_, respectively, with $\alpha ={\left(\alpha_{1}^{\prime },...,\alpha_{7}^{\prime }\right)}^{\prime }$. The dimensions of these matrices correspond to the number of columns of the first matrix where *ρ* acts, times the number of columns of the second matrix where *ρ* acts (i.e., $\tilde{Q}$ has dimension ncol(**R**_1_) × ncol(***x***_3_) = *q*_1_ *q*_2_ × *q*_3_, $\tilde{\Gamma }$ has dimension ncol(**C**_*s*_) × ncol(**C**_*t*_) = *m* × *T*_f_, and so on). Therefore, it holds that $\text{vec}(\tilde{Q})=\beta$, $\text{vec}\left(\tilde{\Gamma }\right)=\gamma_{\text{f}}$, and $\text{vec}\left({\tilde{A}}_{k}\right)=\alpha_{k}$, for *k* = 1, …, 7.

Next consider the matrix cross-products ${\overset{˘}{X}}^{\prime }W\overset{˘}{X}$, ${\overset{˘}{Z}}^{\prime }W\overset{˘}{Z}$, ${\overset{˘}{X}}^{\prime }W\overset{˘}{Z}$, ${\overset{˘}{Z}}^{\prime }W\overset{˘}{X}$ (which is equal to ${\left({\overset{˘}{X}}^{\prime }W\overset{˘}{Z}\right)}^{\prime }$), ${\overset{˘}{X}}^{\prime }Wz$, and ${\overset{˘}{Z}}^{\prime }Wz$ that appear in line 5 in Algorithm 1 (i.e., in the system in Eq (11)). Note first that they can be reduced as:

$$\begin{array}{cc} {\overset{˘}{X}}^{\prime }W\overset{˘}{X} & ={\left(C_{\text{st}}\Gamma X\right)}^{\prime }W^{-1}\left(C_{\text{st}}\Gamma X\right)\text{,}\;{\overset{˘}{Z}}^{\prime }W\overset{˘}{Z}={\left(C_{\text{st}}\Gamma Z\right)}^{\prime }W^{-1}\left(C_{\text{st}}\Gamma Z\right)\text{,}\\ {\overset{˘}{X}}^{\prime }W\overset{˘}{Z} & ={\left(C_{\text{st}}\Gamma X\right)}^{\prime }W^{-1}\left(C_{\text{st}}\Gamma Z\right)\text{,}\;{\overset{˘}{X}}^{\prime }Wz={\left(C_{\text{st}}\Gamma X\right)}^{\prime }z\text{,}\\ {\overset{˘}{Z}}^{\prime }Wz & ={\left(C_{\text{st}}\Gamma Z\right)}^{\prime }z\text{.} \end{array}$$

Therefore, we only need to compute **C**_st_ **Γ** ***x*** and **C**_st_ **Γ** **Z**. These expressions are obtained as follows:

$$\begin{array}{cc} C_{\text{st}}\Gamma X & \equiv \rho (G{\left(X_{3},C_{\text{t}}^{\prime }\right)}^{\prime },\rho \left\{G{\left(R_{1},C_{\text{s}}^{\prime }\right)}^{\prime },\tilde{\Gamma }\right\})\text{,}\\ C_{\text{st}}\Gamma Z & \equiv [\rho \left(G{\left(Z_{3},C_{\text{t}}^{\prime }\right)}^{\prime },\rho \left\{G{\left(R_{1},C_{\text{s}}^{\prime }\right)}^{\prime },\tilde{\Gamma }\right\}\right):\rho \left(G{\left(X_{3},C_{\text{t}}^{\prime }\right)}^{\prime },\rho \left\{G{\left(R_{2},C_{\text{s}}^{\prime }\right)}^{\prime },\tilde{\Gamma }\right\}\right):\\ & \;\;\;\rho \left(G{\left(X_{3},C_{\text{t}}^{\prime }\right)}^{\prime },\rho \left\{G{\left(R_{3},C_{\text{s}}^{\prime }\right)}^{\prime },\tilde{\Gamma }\right\}\right):\rho \left(G{\left(Z_{3},C_{\text{t}}^{\prime }\right)}^{\prime },\rho \left\{G{\left(R_{2},C_{\text{s}}^{\prime }\right)}^{\prime },\tilde{\Gamma }\right\}\right):\\ & \;\;\;\rho \left(G{\left(Z_{3},C_{\text{t}}^{\prime }\right)}^{\prime },\rho \left\{G{\left(R_{3},C_{\text{s}}^{\prime }\right)}^{\prime },\tilde{\Gamma }\right\}\right):\rho \left(G{\left(X_{3},C_{\text{t}}^{\prime }\right)}^{\prime },\rho \left\{G{\left(R_{4},C_{\text{s}}^{\prime }\right)}^{\prime },\tilde{\Gamma }\right\}\right):\\ & \;\;\;\rho (G{\left(Z_{3},C_{\text{t}}^{\prime }\right)}^{\prime },\rho \left\{G{\left(R_{4},C_{\text{s}}^{\prime }\right)}^{\prime },\tilde{\Gamma }\right\})]\text{.} \end{array}$$

## Results

Once the ST-PCLM has been stated, we analyzed the data related to Q fever outbreaks in the Netherlands.

### Weekly high-resolution smooth incidence maps

As mentioned above, we have focused on the distribution of Q fever incidence in the south of the Netherlands during 2009. Some of this data were analyzed in [41], the authors focused on the construction of a smooth incidence map at a fixed time, without considering the need to obtain predictions that were consistent with the aggregated data. More recently [42] focused on the spatial transmission of the disease.

Let’s remember that the data are aggregated at municipality and monthly levels, and that our goal is to obtain estimates of Q fever incidence at that fine grid and at each week of 2009 (i.e., disaggregate simultaneously in space and time) to obtain better insights of the evolution of the disease. The ST-PCLM approach, allows to visualize Q fever incidence at a finer spatio-temporal resolution, and also to incorporate fine-scale population information to the estimation of the latent process. Here we use two sources of information: 1) The Q fever counts that are available in the municipalities of the study area described in Fig 2b and months of 2009 (i.e., the initial spatial and temporal scales are municipalities and months, respectively), and 2) population measured at a fine spatial grid over the study area. Population information at fine spatial grids is available from the WorldPop project. The data containing the spatial distribution of population in 2009 in the Netherlands can be downloaded from https://www.worldpop.org/geodata/summary?id=42722.

**Fig 3:**
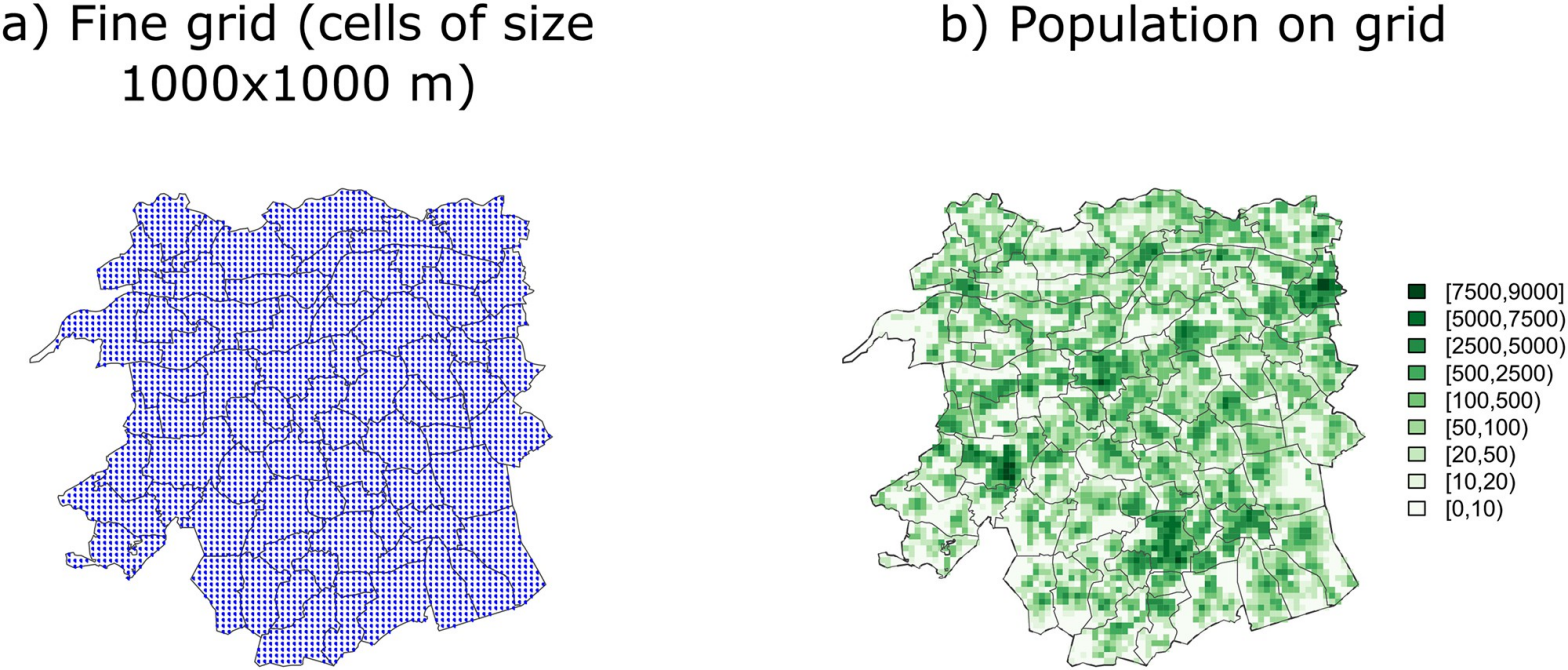
The map on the left shows the fine grid of 1000 × 1000 m cells in the study area shown in Fig 2b. The map on the right shows the spatial distribution of the population on this fine grid. Source: Estimated population density per grid-cell from the WorldPop project in 2009, the Netherlands https://www.worldpop.org/geodata/summary?id=42722. a) Fine grid (cells of size 1000x1000 m). b) Population on grid.

Fig 3a shows a fine grid composed of 4871 regular cells of size 1000 m × 1000 m. Blue dots represent the spatial coordinates of the centroids of these cells, and Fig 3b shows the spatial distribution of the population on this fine grid, which is heterogeneous across municipalities. To set up the ST-PCLM formulation, we have used: centroids of the cells described in Fig 3a as fine-scale spatial coordinates, i.e., as ***x***_1_ and ***x***_2_, ***x***_3_ = (1, …, 53)′ (since 53 weeks were observed in 2009), second order penalties, 12 equally-spaced knots for the marginal cubic B-spline bases **B**_1_ and **B**_2_, and 8 equally-spaced knots for the marginal cubic B-spline basis **B**_3_. We have assumed here that the population at the fine grid is constant throughout the time period; thus, **e**_f_ in model (6) is considered as a vector obtained by repeating the fine-scale population fifty three times. The elements of the spatial composition matrix are obtained using Eq (5), whereas the temporal composition matrix for this case has the following form:

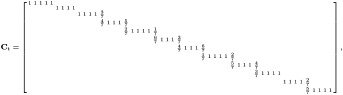

where Sunday is considered the first day of the week. As opposed to the spatial composition matrix, the matrix **C**_*t*_ has some entries that are fractions. This is because some months share parts of a specific week (for example, some days of week 14 belong to March and the others to April).

**Fig 4:**
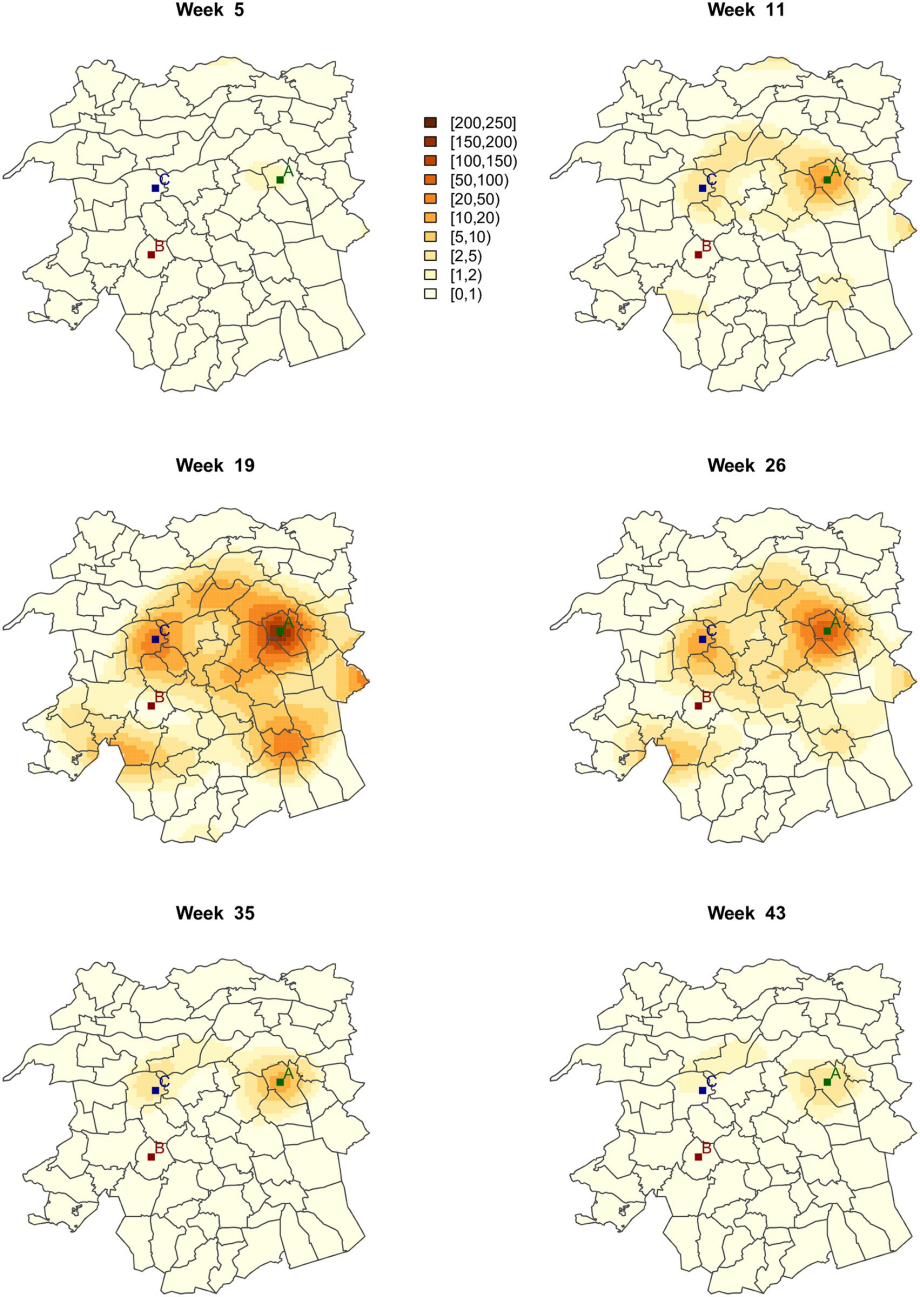
Smoothed Q fever incidence at a detailed spatio-temporal scale, resulting from the ST-PCLM approach, for six selected weeks.

**Fig 5:**
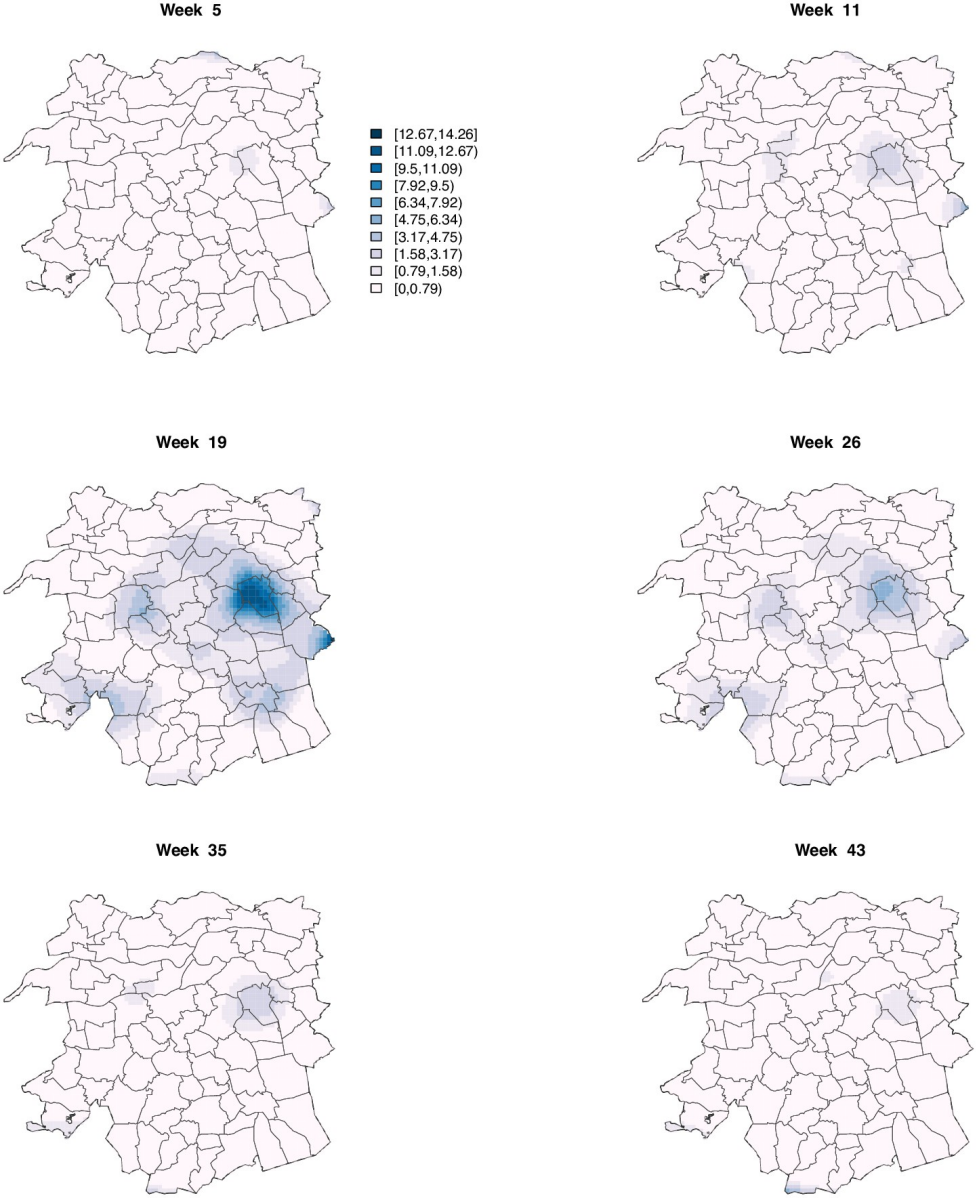
Approximate standard error maps associated with the smoothed Q fever incidence maps in Fig 4.

Fig 4 shows the resulting ST-PCLM Q fever incidence (per 100000 inhabitants) at the target fine spatial resolution, for six selected weeks. These incidences are obtained as $\hat{\text{inc}}=100000\text{exp}\left(X\hat{\beta }+Z\hat{\alpha }\right)$. The evolution of the incidence varies across municipalities and weeks, where the highest incidences are observed mostly around week 19. Most of those weeks belong to April, May, and June, which have the largest number of Q fever outbreaks observed in 2009 (see Fig 1). Note also that most of the highest incidences in week 19 are spatially concentrated around the area that includes points A and C in Fig 4, which are located in the municipalities of Landerd and Heusden, respectively (see Fig 2b). Fig 5 shows the approximate standard error maps associated with Fig 4. As expected in a Poisson setting, larger variances are found in areas with higher incidence rates.

**Fig 6:**
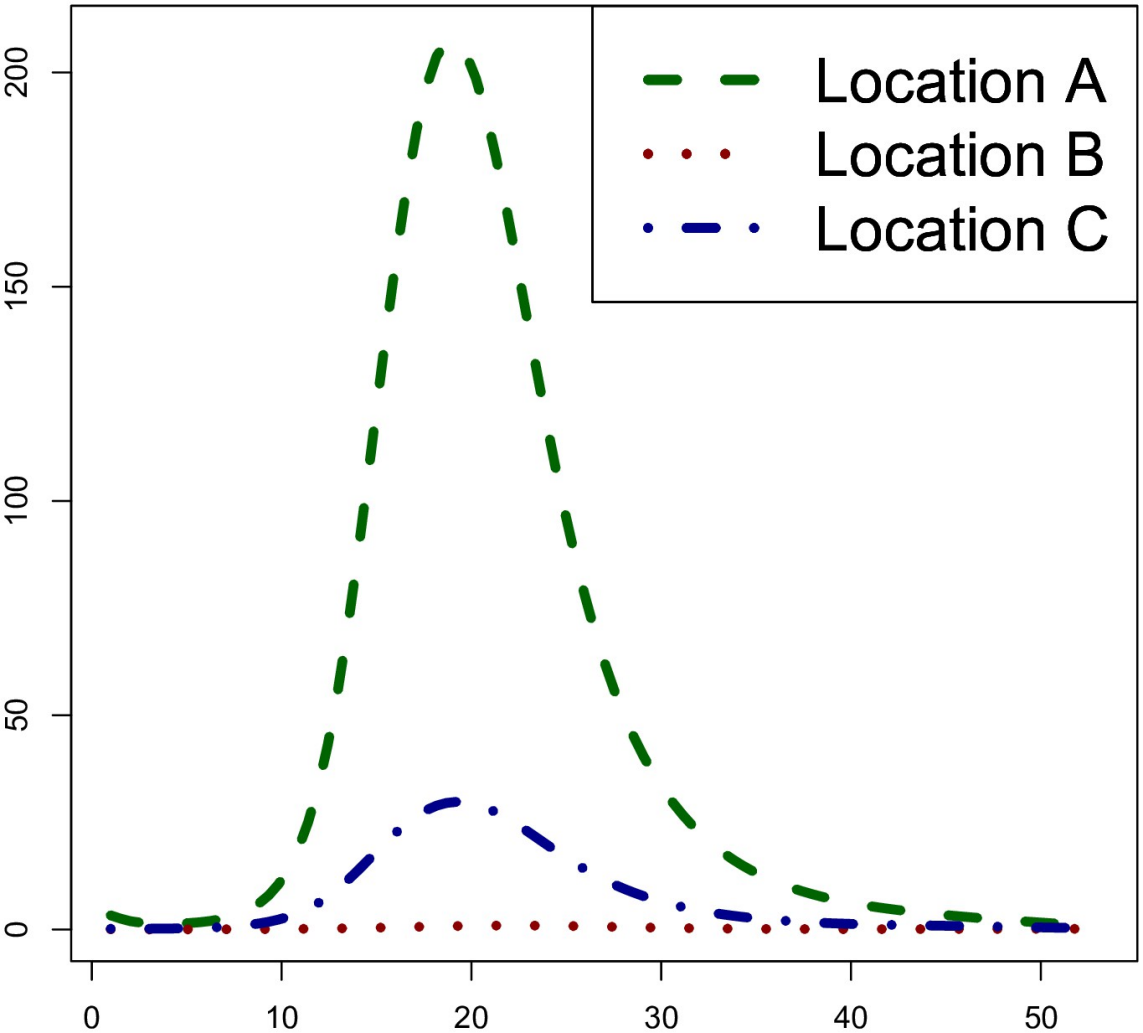
Weekly temporal evolution of Q fever incidence in three specific points (A, B, and C), spatially presented in Fig 4 on the high-resolution map at week 19.

From the previous ST-PCLM estimates, we can also visualize the disaggregated (weekly) temporal evolution of the Q fever disease at specifics spatial coordinates of the fine grid. Fig 6 shows the down-scaled smoothed temporal incidence (per week) at three specific locations A, B, and C, in the study area. We observe that the temporal evolution of the incidence at point B is constant and almost zero, whereas, at points A and C, the temporally smoothed incidence present a unimodal behaviour, where the peak is reached around week 19 (May). This is consistent with the summaries of the incidences given in Figs 1 and 2.

### Simulation studies

In this Section, we present one of two simulation studies that have been carried out to examine the predictive performance of the model under different scenarios. In both of them, we have done spatial and temporal disaggregation, but the focus of each simulation exercise is different. The first aims to check the performance of the ST-PCLM approach under different degrees of spatial dependence. In the second, we will study the behaviour of the model under different levels of temporal disaggregation (results from this second study are available in the S1 File).

#### Simulation study 1

Data are generated using the fine grid in Fig 3a as the spatial region of study, and the 53 weeks in a year as the disaggregated temporal scale. The simulation is conducted as follows:

1. The fine-resolution incidence vector is constructed based on the smoothed Q fever incidences obtained in the previous section. We denote these smoothed incidences as inc(***u***_*k*_), where ***u***_*k*_, *k* = 1, …, *K*, with *K* = 4371 × 53 = 258163, represents the spatio-temporal coordinates at fine resolution. The different levels of spatial dependence are achieved by changing the values of the variance components that control the spatial term in the model, $\tau_{1}^{2}$ and $\tau_{2}^{2}$ (the variance component for the temporal term, $\tau_{3}^{2}$, will remain fixed at the optimal value obtained in the fit). The values for the optimal variance components in the fit were: $\tau_{1}^{2}=218.7$, $\tau_{2}^{2}=127.7$, and $\tau_{3}^{2}=92.4$. Based on these results we set 3 different scenarios:
  - Scenario 1: Variances for the spatial component are those in the fit.
  - Scenario 2: Variances for the spatial component are 100 times larger than those in the fit.
  - Scenario 3: Variance for the spatial component are 1000 times smaller than those in the fit.
2. Calculation of the aggregated expected number of cases (over municipalities and months), *μ*_*kl*_, *k* = 1, …, 72, *l* = 1, …, 12:
  $$\mu =\left(C_{\text{t}}⊗C_{\text{s}}\right)\text{inc}\left(u_{k}\right).$$
3. 100 realizations of the number of cases in each municipality and month are generated through a random drawing from a Poisson distribution with mean parameter ***μ***.

**Fig 7:**
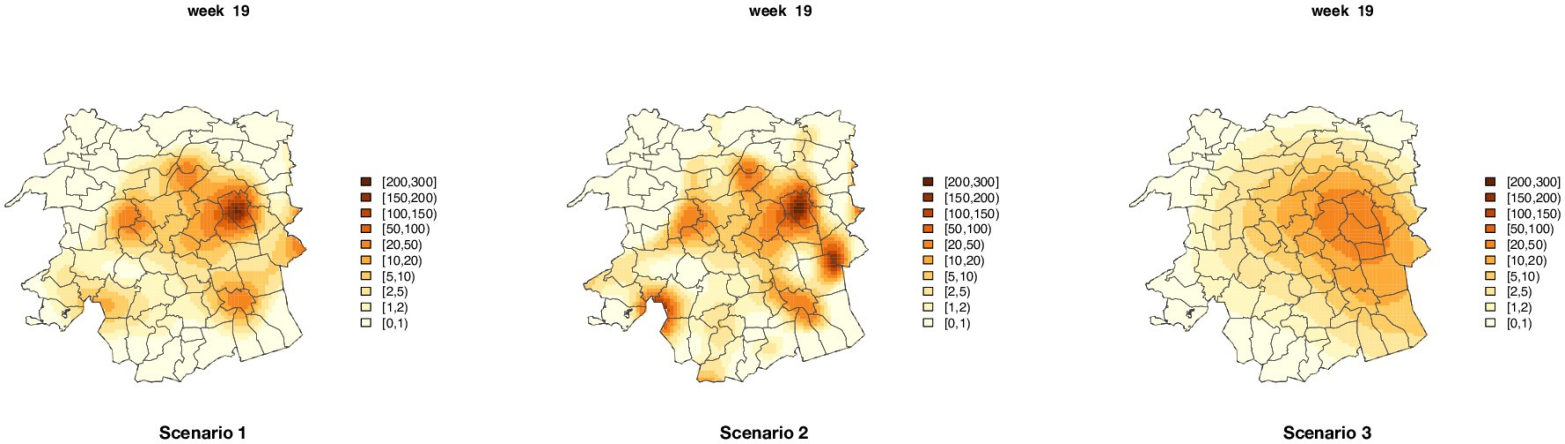
Plots of incidences (for one of the weeks) used in each simulated scenario.

Fig 7 shows the incidences, inc_*g*_(***u***_*k*_), corresponding to week 19 (which is the one with the highest number of cases) used in the three scenarios of simulation study 1. They reflect the three different levels of smoothness obtained by increasing or decreasing the variance components that control the spatial effect in the model.

For all realizations *l* = 1, …, 100, the predicted incidences ${\text{inc}}_{{\text{P}}_{g}}^{\left(l\right)}\left(u_{k}\right)$ obtained from the ST-PCLM approach for each scenario *g*, with *g* = 1, 2, 3, were compared to the smoothed incidences inc_*g*_(***u***_*k*_). To evaluate the performance of the model we computed, for each scenario, the mean absolute error (MAE), the root mean squared error (RMSE):

$$\begin{array}{cc} {\text{MAE}}_{g}^{\left(l\right)} & =\frac{1}{K}\sum_{k=1}^{K}|{\text{inc}}_{{\text{P}}_{g}}^{\left(l\right)}\left(u_{k}\right)-{\text{inc}}_{g}\left(u_{k}\right)|,\\ {\text{RMSE}}_{g}^{\left(l\right)} & =\sqrt{\frac{1}{K}\sum_{k=1}^{K}{\left({\text{inc}}_{{\text{P}}_{g}}^{\left(l\right)}\left(u_{k}\right)-{\text{inc}}_{g}\left(u_{k}\right)\right)}^{2}}, \end{array}$$

the correlation between the observed and predicted incidence, and the percent of grid cells with true incidence falling within 95% prediction intervals of the model. These metrics were averaged over 100 simulated data sets and they are summarized in Table 1.

**Table 1:** Performance comparison of the ST-PCLM approach in three different types of scenarios of simulation study 1. Correlation coefficient average (avg) of the 100 replicates, average coverage % and mean absolute errors (MAE) and root mean squared error (RMSE) are also shown.

Scenarios	Correlation	Coverage	MAE	RMSE
1	0.9829	92.06%	0.00159	0.00996
2	0.9566	89.50%	0.00255	0.01619
3	0.9941	96.44%	0.00086	0.00434

The model performance criteria reported in Table 1 show good model performance in terms of prediction accuracy in all scenarios. All criteria showed that slightly worse predictions where obtained in scenario 2, this is mainly due to the fact that, for consistency, we have used the same size of B-spline basis for all scenarios (those used in the data analysis), but in the case of rapidly changing spatial patters (as is the case in scenario 2), a larger basis would be necessary to correctly capture the spatial effect, or adaptive P-splines [43] could be used; however, this approach is beyond of the scope of this paper.

**Fig 8:**
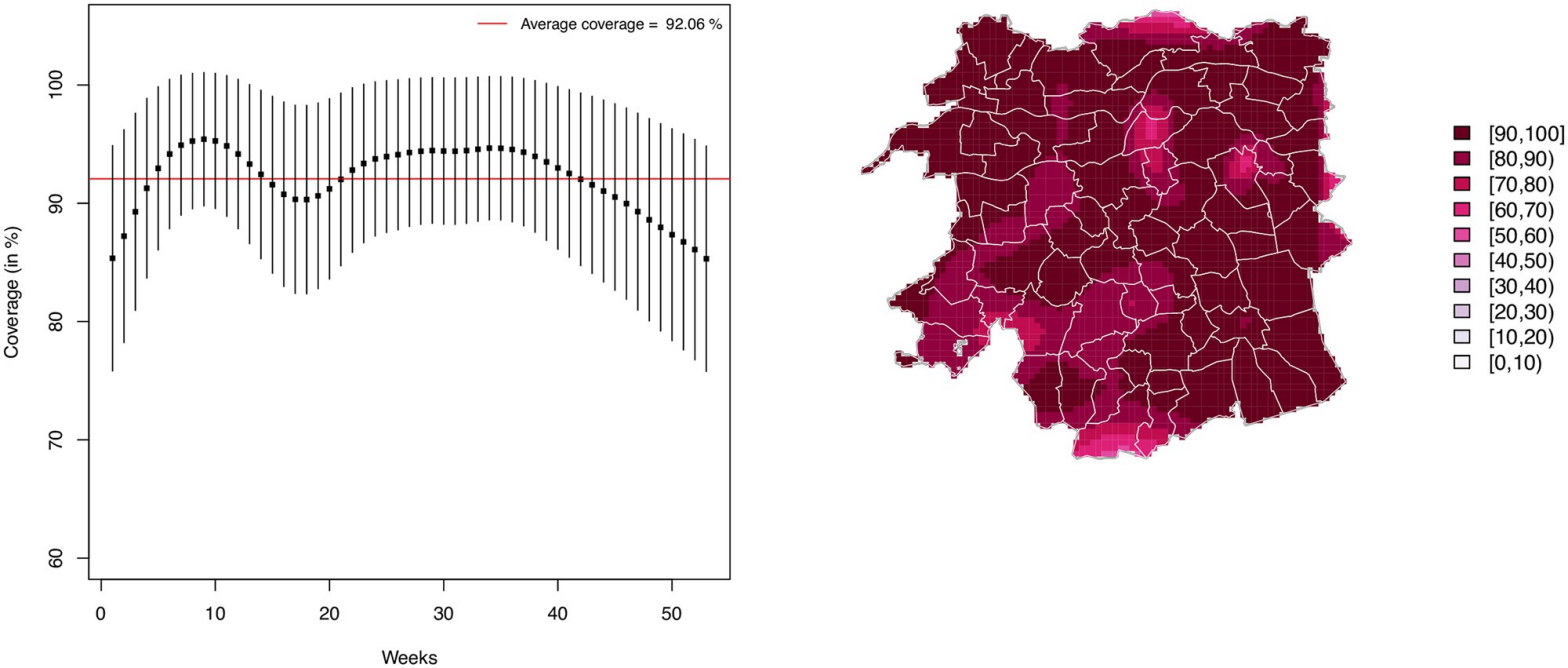
Percentage of cells in the grid with true incidence falling within 95% prediction in scenario 1. On the left, averaged coverage per week over all cells in the grid, and on the right, averaged coverage per cell over all weeks.

In Fig 8 we provide further insight on the 95% coverage achieved in scenario 1 (similar plots for the other scenarios can be found in the S1 File). The lowest coverage over time (averaged over all pixels) was 85%, and was found at the extremes of the time interval (corresponding to the periods of lower incidence). The coverage for each pixel at the fine-grid resolution (averaged over time), shows that areas with lower coverage (60%–80%) correspond to locations with the highest number of cases. However, the coverage for these pixels is not constant over time, reaching almost 100% in weeks with low incidence rates. In scenario 2, the results are similar, although the coverage is lower (especially for a small number of pixels where the averaged coverage is between 20% and 30%), this is mainly due to the poor fitting for points with a sudden high incidence pick. The coverage in scenario 3 (the smoothest spatial trend) is nearly constant over the grid, ranging from 90% to 100%.

Taking into account the fact that we are predicting over more than 4000 grid cells and 53 weeks using aggregated data from 72 municipalities and 12 months, these results indicate a good predictive performance of the model.

## Discussion

We have presented a novel model for the disaggregation of grouped data in both space and time, based on the spatio-temporal penalized composite link model approach. This framework was used to model Q fever counts (recorded in municipalities and months) to obtain Q fever incidence estimates over the fine grid and weeks. The model allows to obtain detailed trends in disease incidence, mortality risks, or any other vital rates at a desirable fine spatio-temporal resolution. Therefore, the resulting ST-PCLM outcomes can be displayed as a dynamic map. It also allows to include population information at a fine resolution in the estimation process. The flexibility of the model is provided by the use of B-splines, along with a penalty on the regression coefficients, and the link between the areas and the fine-resolution grid is achieved through the composition matrix. Our proposal can also address situations in which population information is recorded over small spatial units that are nested in coarser ones (for example, from municipalities to census tracts). In that case, the centroids of the small units can be used to represent the fine spatial scale.

Furthermore, the model allows the incorporation of covariates of interest (such as, for example, socio-economic, demographic, and environmental factors) in the ST-PCLM formulation to improve the estimation of the latent trend. They can be included at the aggregated level or at the fine-scale level (or at both levels simultaneously). The inclusion of covariates at the fine-scale level is immediate by adding them as columns in the design matrix **X** in (6) (if a linear relationship is assumed), or adding columns in **Z** if a non-linear relationship is expected. Details on how to include explanatory variables measured at the aggregated level can be found in [20].

It is important to acknowledge the use of GLAM methods in conjunction with the SAP algorithm, to avoid storage problems and to speed up computations. However, we are aware that the disaggregation of grouped data into a very detailed resolution could lead to increased computational load and storage problems. The sparsity of the marginal composition matrices can be exploited to deal with these issues (see, for example, [44]).

We have conducted two simulation studies to evaluate the prediction accuracy of the ST-PCLM. The first study aimed to check the performance of the model under different degrees of spatial dependence, and the second to asses the effect of different levels of temporal disaggregation. The overall performance of the model was good, although, as expected, it was affected by the number of units available at the aggregated level.

In the ST-PCLM formulation, we assume the spatial unit boundaries remain fixed over time. But it may happen that some of these boundaries change over the years. This issue is known in the statistical literature as the *spatio-temporal misalignment problem*. As future work, we plan to extend the ST-PCLM approach to handle this problem, where marginal composition matrices will play an important role. Furthermore, we can exploit the unique relationship between the penalties associated with basis and the covariance structure they yield to explore the use of other common spatio-temporal covariance matrices.

The implementation of our proposal and all data analyses were carried out using the statistical software R [45].

## Supporting information

S1 File (PDF)
