# Supplementary material for: Modeling latent spatio-temporal disease incidence using penalized composite link models
Source: PLoS One. 2022 Mar 10;17(3):e0263711. doi: 10.1371/journal.pone.0263711 (PMC8912133; doi:10.1371/journal.pone.0263711)
Supplement: S1 File — (PDF) [file pone.0263711.s001.pdf]

Supporting information for: "Modeling latent spatio-temporal disease incidence using penalized composite link models" by D-J. Lee, Durban, M., D. Ayma, and J. Van de Kassteele

September 30, 2021

A: GLAM algebraic operations

In this section we introduced some notation and definitions of array methods proposed in Currie et al. (2006) and Eilers et al. (2006).

Definition A.1. The row tensor of matrices $\mathbf{X_1}$ and $\mathbf{X_2}$ of dimensions $n \times c_1$ and $n \times c_2$, respectively is defined as:

$$\mathcal{G}(\mathbf{X_1}, \mathbf{X_2}) = (\mathbf{X_1} \otimes \mathbf{1}'_{c_2}) \odot \mathbf{1}'_{c_1} \otimes \mathbf{X_2})$$

Definition A.2. The $\mathcal{H}$-transform of the d-dimensional array $\mathbf{A}$ of size $c_1 \times c_2 \times ... \times c_d$ by the matrix $\mathbf{X}$ of size $r \times c_1$ is denoted $\mathcal{H}(\mathbf{X}, \mathbf{A})$ and defined as: let $\mathbf{A}^*$ of size $c_1 \times c_2c_3...c_d$ the matrix obtained by flattering dimensions 2 to d of $\mathbf{A}$; form the matrix product $\mathbf{XA}^*$ of size $r \times c_2c_3...c_d$; then $\mathcal{H}(\mathbf{X}, \mathbf{A})$ is the d-dimensional array of size $r \times c_2 \times ... \times c_d$ obtained from $\mathbf{XA}^*$ by reinstating dimensions 2 to d of $\mathbf{A}$.

If $\mathbf{A}$ is a vector $\mathbf{a}$, we have that $\mathcal{H}(\mathbf{X}, \mathbf{a}) = \mathbf{Xa}$, while if $\mathbf{A}$ ia a matrix, $\mathcal{H}(\mathbf{X}, \mathbf{A}) = \mathbf{XA}$. Thus, the $\mathcal{H}$-transform generalizes premultiplication of vectors and matrices by a matrix.

Definition A.3. The rotation of the d-dimensional array $\mathbf{A}$ of size $c_1 \times c_2 \cdots c_d$ is the d-dimensional array $R(\mathbf{A})$ of size $c_2 \times c_3 \cdots c_d \times c_1$ obtained by permuting the indices of $\mathbf{A}$.

Definition A.4. The rotated $\mathcal{H}$-transform of the array $\mathbf{A}$ by the matrix $\mathbf{X}$ is given by

$$\rho(\mathbf{X}, \mathbf{A}) = \mathcal{R}(\mathcal{H}(\mathbf{X}, \mathbf{A}))$$

B: Graphical displays for scenarios 2 and 3 in simulation study 1

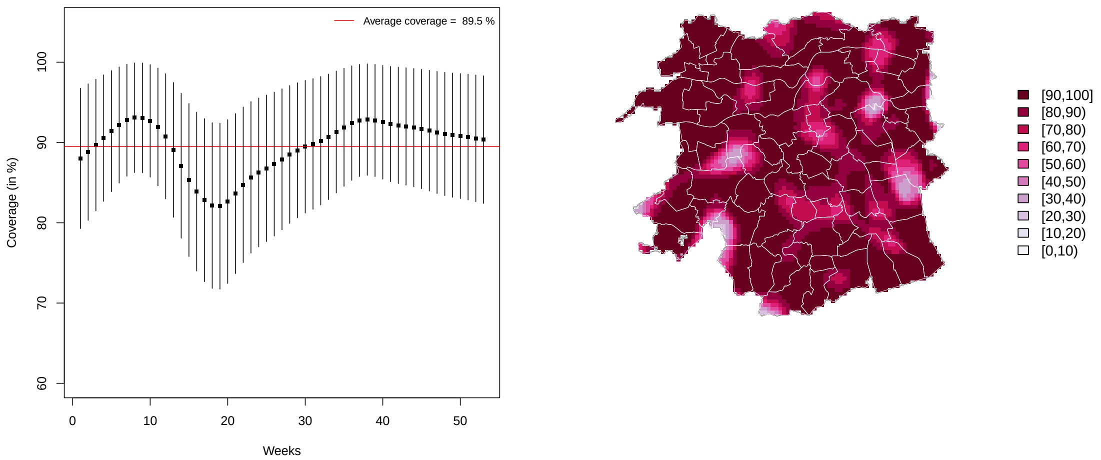

S1 Fig 1. Coverage plots of scenario 2 in simulation study 1. Percent of grid cells with true incidence falling within 95% prediction in scenario 2. On the left, averaged coverage per week over all cell in the grid, and on the right, averaged coverage per cell over all weeks

C: Simulation study 2

In this Section we perform a simulation study in order to examine the prediction performance of the SCLM approach described in the paper . For that we use a detailed dataset, where the full postal code of the home address and the date of onset of illness of the patients suffering from acute Q fever in the Netherlands, 2009, are available. This dataset is publicly restricted due to confidentiality reasons, and it is only used here to asses how well the SCLM approach recovers the true latent Q fever incidence when data are available at a specific coarse scale.

Using the full postal code of home address of patients, we can determine the number of cases occurred per week and in each cell of the fine grid depicted in Figure 3a. Thus, using the population on the fine grid depicted in Figure 3b (and assuming that is constant over weeks), we can obtain a smoothed Q fever incidence surface at this fine spatio-temporal scale using the PGLM approach, where the centroids of the cells described in Figure 3a are used as fine-scale spatial coordinates, and the number of weeks is used as temporal coordinates. This smoothed incidence surface is considered here as the true latent incidence trend at the fine resolution. We denote these smoothed incidences as $\text{inc}(\boldsymbol{u}_k)$, where $\boldsymbol{u}_k$, $k = 1, ..., K$, with $K = 4371 \cdot 53 = 258163$, represents the spatio-temporal coordinates at

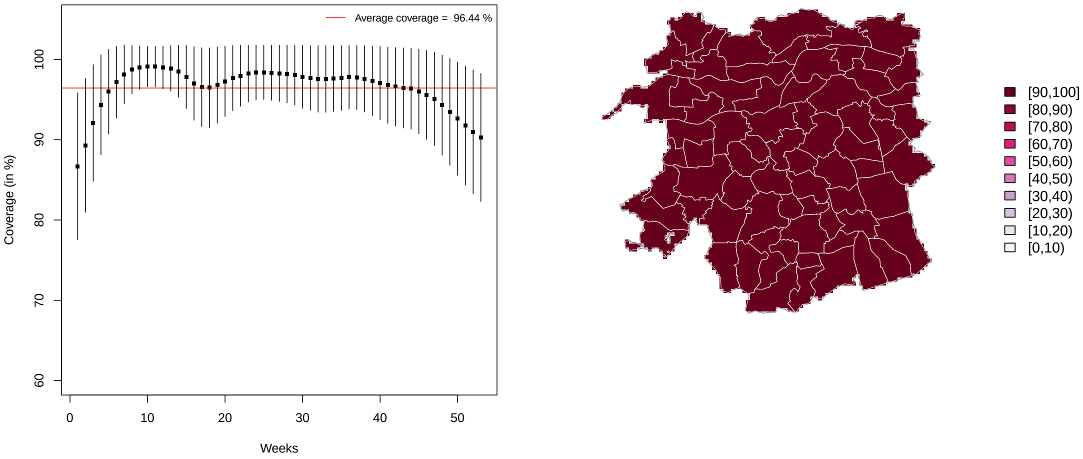

S1 Fig 2. Coverage plots of scenario 3 in simulation study 1. Percent of grid cells with true incidence falling within 95% prediction in scenario 3. On the left, averaged coverage per week over all cell in the grid, and on the right, averaged coverage per cell over all weeks

fine resolution. To study the prediction performance of the SCLM approach, we artificially aggregate these Q fever incidence estimates by municipalities and by several coarse periods of time.

The simulation study was conducted as follows:

(a) The fine-scale smoothed incidences described above and the population on the fine grid depicted in Figure3a (assumed constant over weeks) were used to calculate the Q fever incidence for each municipality v_i, $i = 1, ..., 72$ (which are depicted in Figure 3b), and in different coarse temporal resolutions: 1) fortnights (two weeks); 2) months; and 3) bimesters (two months). Thus we have three types of spatio-temporal aggregation: $g = 1$ where data are summarized over municipalities and fortnights, $g = 2$ where data are summarized over municipalities and months, and $g = 3$ where data are summarized over municipalities and bimesters.

(b) 100 realizations of the number of cases recorded over municipalities and each coarse temporal resolution were generated by random drawing of a Poisson distribution whose mean parameter is calculated as the corresponding aggregated incidence (obtained in the previous step) times the population recorded at the appropriate coarse resolution.

(c) For each realization, we apply the SCLM approach using the population on the fine grid (repeated 53 times) as the vector $\boldsymbol{e}_{\mathrm{f}}$.

For all $l = 1, ..., 100$ realizations, the predicted incidence $\mathrm{inc}^{(l)}_{\mathrm{P}_g}(\boldsymbol{u}_k)$ obtained from the

SCLM approach of each type of aggregation g, with $g = 1, 2, 3$, were compared to the smoothed incidences $\text{inc}(\boldsymbol{u}_k)$, $k = 1, ..., K$, using the mean absolute error (MAE) and the root mean squared error (RMSE) criteria defined as:

$$\text{MAE}_g^{(l)} = \frac{1}{K} \sum_{k=1}^{K} \left| \text{inc}_{\text{P}_g}^{(l)}(\boldsymbol{u}_k) - \text{inc}(\boldsymbol{u}_k) \right|$$

$$\text{RMSE}_g^{(l)} = \sqrt{\frac{1}{K} \sum_{k=1}^{K} \left(\text{inc}_{\text{P}_g}^{(l)}(\boldsymbol{u}_k) - \text{inc}(\boldsymbol{u}_k) \right)^2}$$

Due to the confidentiality of the data used to carry out the simulations, we summarize the results in S2 Table 2 that displays the averages and the standard deviations of the resulting errors (for each criterion) derived from the simulation study. As we could expect we have found the SCLMs estimates that were obtained from the most coarse spatio-temporal aggregation (type of aggregation 3) are less similar to the true incidences. Note that these are overall results, and the spatial support remains the same in all type of aggregations (municipalities). Similar performances as in S2 Table 2 are obtained if the errors are analyzed by weeks. The behavior of the different area of the map is consistent across the levels of aggregation, i.e., grid cells with high/low RMSE are located in similar areas in the case of monthly, bimester or fortnight aggregation.

	MAE		RMSE	
Type of aggregation	avg	std	avg	std
$g = 1$: fortnights	0.3856	0.0321	1.3413	0.2774
$g = 2$: months	0.3935	0.0276	1.3933	0.2327
$g = 3$: bimesters	0.4142	0.0326	1.4625	0.2469

MAE: mean squared error; *RMSE:* root mean squared error; *avg:* average; *std:* standard deviation.

S1 Table 1. Comparison of results of simulation study 2. Performance comparison of the SCLM approach under three different types of aggregation (1: municipalities and fortnights; 2: municipalities and months; 3: municipalities and bimesters), using different criteria: mean absolute errors and root mean squared errors. These errors are summarized in terms of the average (avg) and standard deviation (std).

References

Currie ID, Durbán M, Eilers PHC. Generalized linear array models with applications to multidimensional smoothing. J R Statist Soc B. 2006;68(2):259–280.

Eilers PHC, Currie ID, Durbán M. Fast and compact smoothing on large multidimensional grids. Computational Statistics & Data Analysis. 2006;50(1):61–76.
